# Ten Years after the *Prestige* Oil Spill: Seabird Trophic Ecology as Indicator of Long-Term Effects on the Coastal Marine Ecosystem

**DOI:** 10.1371/journal.pone.0077360

**Published:** 2013-10-09

**Authors:** Rocío Moreno, Lluís Jover, Carmen Diez, Francesc Sardà, Carola Sanpera

**Affiliations:** 1 Department Biologia Animal, Facultat de Biologia, Universitat de Barcelona, Barcelona, Spain; 2 Department Salut Pública, Facultat de Medicina, Universitat de Barcelona, Barcelona, Spain; 3 Department Ecoloxía e Bioloxía Animal. Facultade de Ciencias, Universidade de Vigo, Galicia, Spain; 4 Institut Català d’Ornitologia (ICO), Museu de Zoologia, Barcelona, Spain; Àrea de Biodiversitat, Centre Tecnològic Forestal de Catalunya (CTFC), Solsona, Spain; 5 Department Biologia Animal, Facultat de Biologia, Universitat de Barcelona, Barcelona, Spain; National Institute of Water & Atmospheric Research, New Zealand

## Abstract

Major oil spills can have long-term impacts since oil pollution does not only result in acute mortality of marine organisms, but also affects productivity levels, predator-prey dynamics, and damages habitats that support marine communities. However, despite the conservation implications of oil accidents, the monitoring and assessment of its lasting impacts still remains a difficult and daunting task. Here, we used European shags to evaluate the overall, lasting effects of the *Prestige* oil spill (2002) on the affected marine ecosystem. Using *δ* ^15^N and Hg analysis, we trace temporal changes in feeding ecology potentially related to alterations of the food web due to the spill. Using climatic and oceanic data, we also investigate the influence of North Atlantic Oscillation (NAO) index, the sea surface temperature (SST) and the chlorophyll a (Chl a) on the observed changes. Analysis of *δ* ^15^N and Hg concentrations revealed that after the *Prestige* oil spill, shag chicks abruptly switched their trophic level from a diet based on a high percentage of demersal-benthic fish to a higher proportion of pelagic/semi-pelagic species. There was no evidence that Chl a, SST and NAO reflected any particular changes or severity in environmental conditions for any year or season that may explain the sudden change observed in trophic level. Thus, this study highlighted an impact on the marine food web for at least three years. Our results provide the best evidence to date of the long-term consequences of the *Prestig*e oil spill. They also show how, regardless of wider oceanographic variability, lasting impacts on predator-prey dynamics can be assessed using biochemical markers. This is particularly useful if larger scale and longer term monitoring of all trophic levels is unfeasible due to limited funding or high ecosystem complexity.

## Introduction

The marine environment is exposed to a wide range of anthropogenic impacts that result in complex and still largely unknown adverse effects on marine populations and whole ecosystems. Recently, the *Deepwater Horizon* oil spill in the Gulf of Mexico emphasized the necessity of achieving a wide understanding of long-term effects to predict the fate of affected ecosystems and to choose appropriate monitoring and restoration policies [1–3]. Even though the amount of oil spilled into the oceans has increased in recent years, for less than 20 of the 100 documented large spills did monitoring of effects continue for greater than 5 years [4]. It was not until the 1989 *Exxon Valdez* oil spill that the largest investment in oil pollution research to date gave rise to an evaluation of ecological impacts of unprecedented scope and duration [5,6]. Such research efforts highlighted that oil persisted beyond a decade in surprising amounts and in toxic forms, and that acute pollution events could have long-term impacts at the population level [5]. Similarly, four decades after the 1969 *Florida* oil spill, the lingering effects on large-scale ecosystem functions were still evident [7]. Although recovery from oil spills depends on the spill type and on local environmental characteristics [8], these findings not only cast doubt on the old paradigms of “rapid recovery”, but also provided a new understanding of the biological effects of petroleum: major oil spills can have long-term impacts since oil pollution does not only result in acute mortality of marine organisms, but also affects productivity levels and predator-prey dynamics, and damages habitats that support marine communities.

Monitoring such lasting consequences, however, can be a daunting task. Major oil spills always get substantial public and government attention in the first few months, but interest quickly tails off when oil and dead animals are removed from the public eye. As a consequence, although injury to the environment remains, funding to assess long-term indirect effects becomes increasingly scarce. Moreover, confounding factors such as natural environmental variation or the lack of pre-spill data for most of the marine ecosystems may hamper attempts to assess the impacts, mask the effects or lead to contradictory assessments [9–11].

Upper trophic level predators such as seabirds have been shown to be reliable indicators of oil spill impacts in spite of the influence of environmental factors [11]. Also, feathers formed before the oil spill obtained from seabird corpses offer a unique opportunity to compile pre-spill data on feeding habitat and diet [12]. Moreover, given that seabird feeding ecology reflect alterations in food web trophodynamics [13–17], monitoring changes in their diet allows an assessment of ecosystem status when collection of data from other trophic levels is unfeasible. The discharge of tons of petroleum may cause shifts in the food web through degradation of habitat, population declines, cascading trophic interactions, or changes in behaviour of fish, crustaceans and other organisms [5,18]. In this regard, long-term monitoring studies of seabird feeding ecology have the potential to provide insights into alterations of food web dynamics due to oil-spill contamination and reflect quality status of affected ecosystems.

Although some research programmes to evaluate changes in feeding ecology have been carried out using traditional methods such as analysis of pellets, spontaneous regurgitates or direct observations of items fed to chicks [16,19–21], this sampling represents single, limited “snapshots” of the most recent diet and can be subject to various biases [22]. Alternatively, the analysis of stable isotope ratios in consumer tissues and potential prey offer a robust method to infer assimilated and not only ingested food, and together with other reliable indicators of diet such as mercury concentrations (Hg), have been successfully applied to feeding ecology studies [23–27].

In November 2002, the tanker *Prestige* was wrecked off the Atlantic north-west coast of Spain (Galicia), releasing approximately 60000 tonnes of oil products in one of the major regional oil spill hotspots world-wide [28] and contaminating key marine ecosystems such as the National Park of the Galician Atlantic Islands. However, ten years later and despite the *Prestige* accident was the largest catastrophe of its kind ever recorded in European waters, only one previous study has focused on its long-term ecological impacts [29].

Aiming to evaluate the overall lasting effects of the *Prestige* oil spill on the marine ecosystem, we used as an indicator of the ecosystem health, the European shag, an important member of the nearshore community affected by the wreck [30–32]. With the purpose of tracing temporal changes in its feeding ecology due to the *Prestige* spill, we combined *δ* ^15^N and Hg concentrations of feathers sampled at three affected colonies in the National Park of the Galician Atlantic Islands during six post-spill years with pre-spill data obtained from feathers of dead juveniles collected during the actual accident [12]. In order to be able to compare and appropriately interpret the temporal data, we also considered δ ^15^N and Hg concentrations of potential prey [27] and assessed temporal variation in baseline values. Lastly, since oceanographic and climatic changes have already been demonstrated to influence fish species abundance variation [33–36], we also investigated the potential influence of the North Atlantic Oscillation (NAO) index, sea surface temperature (SST) and chlorophyll a (Chl a) on the observed changes of the biogeochemical markers considered.

## Material and Methods

### Ethic Statement

The three colonies that were sampled are within the National Park of the Galician Atlantic Islands. The National Park issued the permit for sampling of chick feathers of European Shag (*Phalacrocorax aristotelis*), and whole mussels (*Mytilus galloprovincialis*). The sampling was also approved by the Conselleria Medio Ambiente ("Xunta de Galicia" autonomous regional government). In Spain, if laboratory experiments are not involved, these institutions decide on ethical matters related to the sampling of wildlife. The sampling methods were straightforward; chicks in the nest were handled briefly to remove 5 feathers, and researcher spent a minimal time in the colony in order to reduce disturbance.

### Study Area and sampling design

**Figure 1:**
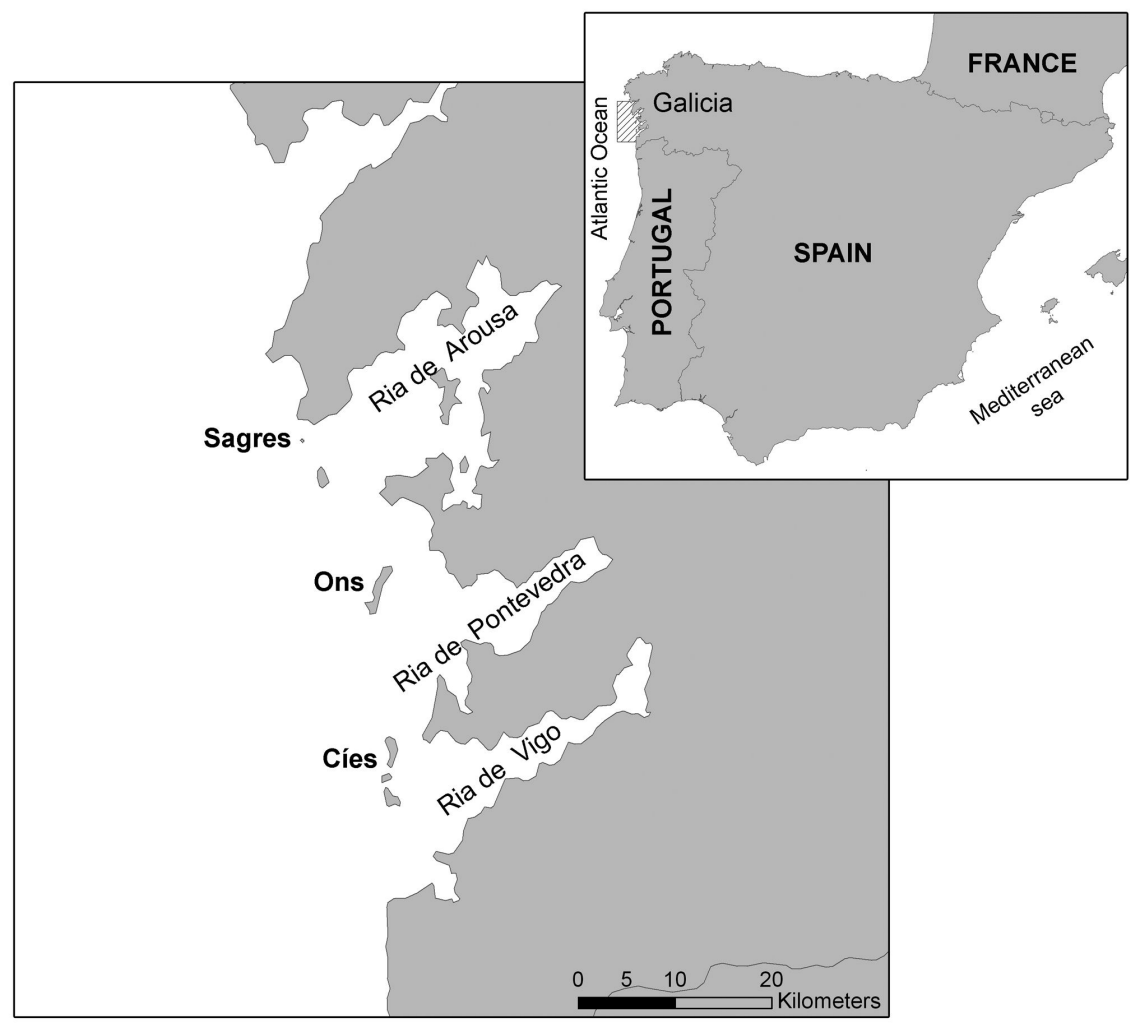
Geographical location of the European shag (*Phalacrocorax aristotelis*) breeding colonies included in this study. The grey rectangle, enlarged in the succeeding image, delimitates the area for which NAO index, SST and Chl a have been provided.

The study area is located on the southern coast of Galicia (NW Spain -Fig. 1-), which is part of the Iberian Coastal Large Marine Ecosystem and corresponds to the area affected by the *Prestige* fuel. Post-spill data were obtained from chick feathers of European shag sampled during the breeding seasons from 2004 to 2009 on three oil-affected colonies (Cíes -n=79-, Ons -n=47- and Sagres -n=53-) that experience the same regional changes in climate and oceanographic conditions [37–39]. Data prior to the spill was gathered from feathers – grown in nest during summer 2002- of corpses of first-year juvenile shags collected during the actual accident in the winter 2002-2003 at the Ría de Vigo [12]. Since chicks from the three colonies have reflected similar feeding habits [27] and showed a similar temporal trend (this article), we considered that pre-spill values obtained from corpses collected at the Ría de Vigo were a valid reference for all colonies.

**Figure 2:**
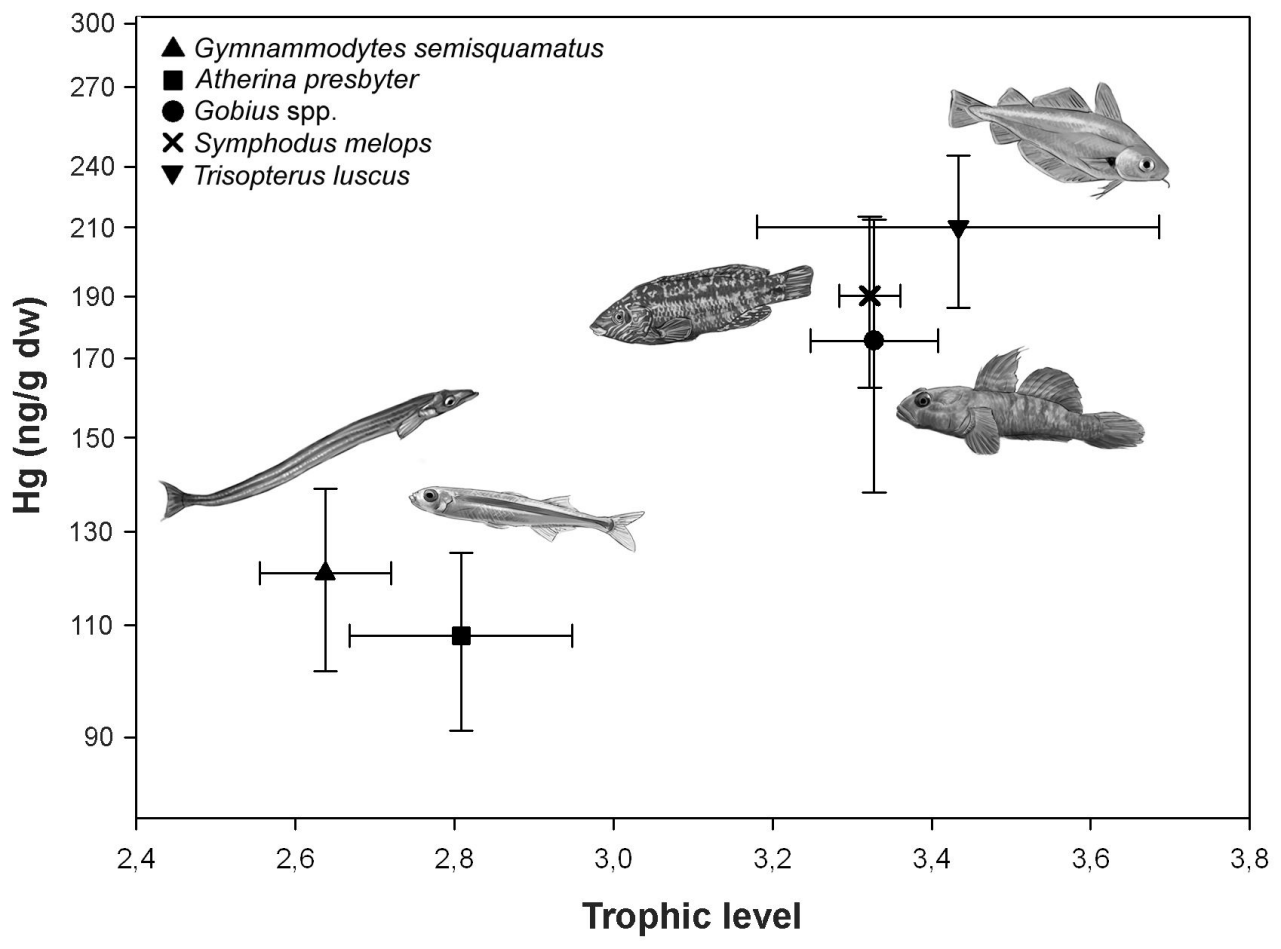
Plot of Hg concentrations (ng/g dry weight in logarithmic scale) and trophic position (mean ± S.D.) in potential prey of European shags sampled in the study area **[27]**. Draws are courtesy from Martin Franch Rodriguez.

To properly interpret Hg and isotopic values obtained from pre- and post-spill feathers, we drew on previously published Hg concentrations and isotopic signatures of potential prey (Figure 2) and their influence on shag isotopic signatures [27]. Because upwelling nitrate from deep oxygen-depleted water can elevate *δ* ^15^N values of marine organisms [40] and the study area is directly influenced by a coastal upwelling, we also collected mussels (*Mytilus galloprovincialis*) during the sampling period as a representative isotopic baseline of the area [27] in order to be able to compare *δ* ^15^N signatures across years [41]. Given that δ^15^N of mussels didn’t vary more than 0.6‰ among years and there was no evidence that Chl a, SST or NAO changes (this article), we used a mean of all values as isotopic baseline of 2002. To test environmental variability over time, we use the NAO index based on the difference of normalized sea level pressure between Ponta Delgada, Azores and Stykkisholmur/Reykjavik, Iceland [42]. Sea MODIS-derived chlorophyll concentrations (mg/m^3^) and SST (°C) were calculated for waters adjacent to colonies (at 42.8° N to 42.0° S and 9.3° W to 8.5° E) with a temporal resolution of 3 months (seasonal) and a spatial resolution of 4 km (http://oceancolor.gsfc.nasa.gov/).

### Stable Isotope Analysis

Feathers were cleaned in a solution of Na OH (0.25M), oven dried at 60°C and kept in polyethylene bags until analysis. For mussels, soft tissues were freeze-dried and lipid extraction was carried out using several chloroform-methanol (2:1) rinses [43] before analysis. To homogenize feathers for stable isotope analysis, we ground them to an extremely fine powder using an impactor mill (Freezer/mill 6750 –Spex Certiprep-) operating at liquid nitrogen temperature. Weighed sub-samples of the powdered feathers and mussels (ca. 0.36 mg) were placed into tin buckets and crimped for combustion. Isotopic analyses were carried out by EA-IRMS (elemental analysis-isotope ratio mass spectrometry) by means of a ThermoFinnigan Flash 1112 coupled to a Delta isotope ratio mass spectrometer via a CONFLOIII interface. Analyses were done at the Serveis Científico-Tècnics of the University of Barcelona.

Nitrogen stable isotope ratios were expressed in conventional notation as parts per thousand (‰), using the following equation:

*δX* = [(*R*_sample_/*R*_standard_) - 1] × 1000

where *X* is ^15^N and *R* is the corresponding ratio ^15^N / ^14^N.

The standard for ^15^N is atmospheric N_2_ (Air). International standards (IAEA) were inserted every 12 samples to calibrate the system and compensate for any drift over time. Precision and accuracy for *δ* ^15^N measurements was ≤0.3.

To compare the *δ* ^15^N signature of chicks across years, we computed their trophic position using the following formula:

Trophic position _consumer_ = λ + (*δ* ^15^N _consumer_ -*δ* ^15^N_base_) / *Δ* ^15^N

where λ is the trophic position of the organism used to estimate *δ* ^15^N_base_ (e.g., λ = 2 for primary consumers such as mussels [44]), *δ* ^15^N_consumer_ is measured directly, and *Δ* ^15^N is the isotopic discrimination factor, the enrichment in *δ* ^15^N per trophic level (we used a mean of 3‰ derived from the literature [45]).

### Mercury analysis

Chemical determination of Hg was carried out by means of ICP-OES, PerkinElmer Elan 6000 (Serveis Científico-Tècnics, University of Barcelona). We digested feather samples (ca. 100 mg) in Teflon TM containers using HNO_3_ (1–2 ml) and H_2_O_2_ (0.5–1 ml) during 14 h at 90°C.

The accuracy of the analysis was checked by measuring certified reference tissue of Human Hair (BCR 397). Mean recoveries ranged 80-92% and no corrections were done.

### Statistical analysis

We routinely checked the values of stable isotope ratios and Hg concentrations for normality using Q-Q plots. Hg concentrations showed skewed distributions and a logarithmic transformation was applied. A general linear model was used to analyze trophic level considering the effects of colony and year. The same modelling approach was used to model log mercury concentration by colony, year and trophic level effects. Colony and year were introduced as factors and trophic level as a continuous covariate. In both cases and in order to assess if environmental conditions prevailing in the area could explain differences between years, we used alternative models replacing the year factor by Chl a, SST and NAO indexes of different seasons (current breeding season and previous autumn, winter and spring). Model selection was made using AICc, Akaike’s Information Criterion adjusted for sample size [46]. To evaluate factors in the model, chi-square likelihood ratio tests (LR) are presented and a posteriori pairwise comparisons were made using sequential Sidak procedure. Statistical analysis was carried out using SPSS (PASW 18.0).

## Results

**Figure 3:**
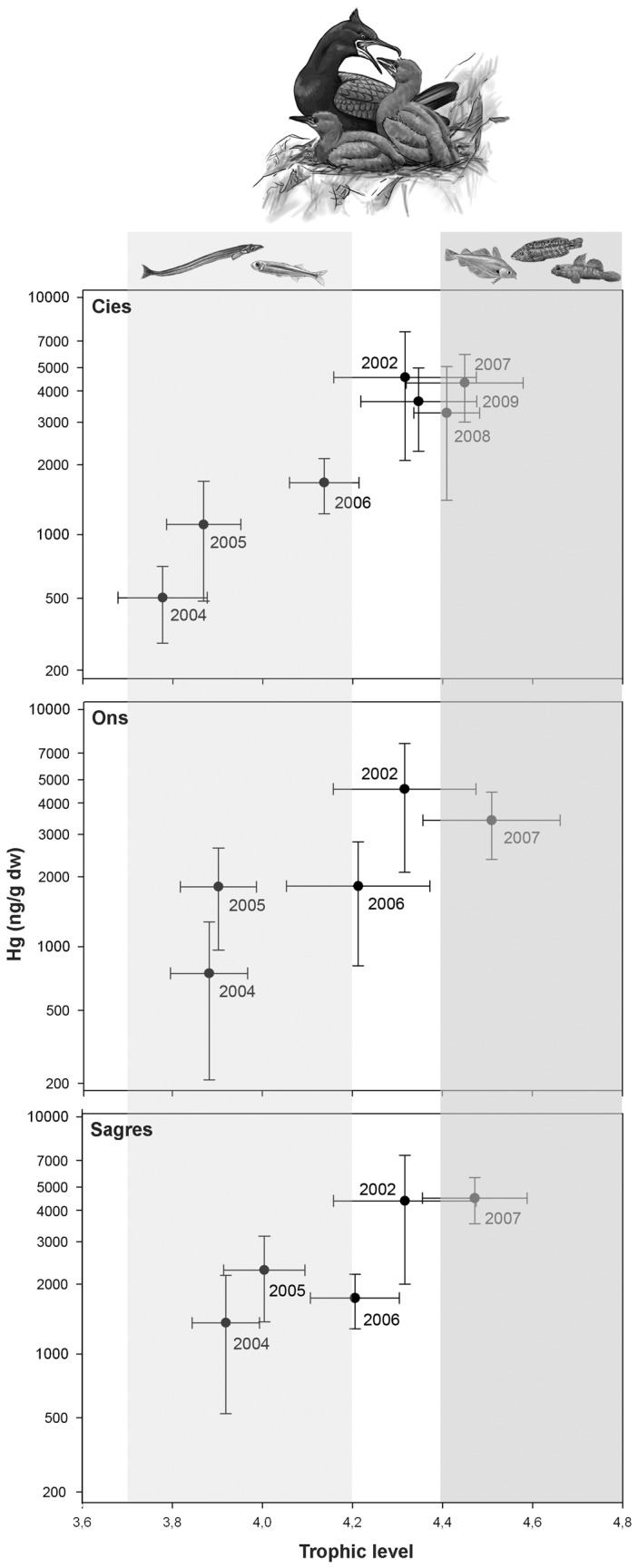
Plot of Hg concentrations (ng/g dry weight in logarithmic scale) and trophic position (mean ± S.D.) in feathers of European shag chicks sampled at three affected colonies from 2004 to 2009 and in feathers from juvenile carcasses collected during the *Prestige* accident representing pre-spill values from 2002, reflecting the diet change due to the wreck. *Light* *grey* *box*: chicks with values corresponding to a diet on a higher percentage of pelagic/semipelagic species. *Dark* *grey* *box*: chicks with values corresponding to a diet on a higher percentage of demersal and benthic fish. Draws are courtesy from Martin Franch Rodriguez.

δ ^15^N and Hg concentrations in feathers of European shags sampled in Cíes, Ons and Sagres from 2002 to 2009 are showed in Table 1. The model selected for trophic level included both colony and year effects (Table 2) without any significant interaction. The colony effect (LR=24.5, d.f.=2, P<0.0001) indicated that chicks from Cíes were feeding at a significantly lower trophic level than those from Ons and Sagres, and that these last two colonies were very close in terms of trophic level. Sampling year was the most important factor (LR=348.9, d.f=6, P <0.0001), showing a temporal trend that indicated a significant and rapid decrease in trophic level after the *Prestige* oil spill, a successive and significant increase throughout 2004 to 2007, and similar values during the 2007-2009 breeding seasons, which, in turn, were not significantly different from the mean value recorded in 2002 (Figure 3).

**Table 1:** Sample sizes and descriptive statistics of *δ* ^15^N (‰) and Hg concentrations (ng/g in dry weight) in feathers of European shag chicks sampled at three affected colonies from 2004 to 2009 and in feathers from juvenile carcasses collected during the *Prestige* accident representing pre-spill values from 2002.

				**Hg(ng/g)**		***δ*^15^N(‰)**
**Colony**	**Year**	**N**	**mean±S.D**	**median**	**min-max**	**mean±S.D**
Cíes	2002	10	3893.6±2090.2	3742.3	899.0-7765.8	14.5±0.4
	2004	20	543.0±193.6	505.2	293.9-1054.9	13.1±0.3
	2005	12	1103.9±578.5	1012.6	369.4-2307.7	13.4±0.2
	2006	14	1658.1±431.9	1603.0	922.2-2396.7	14.2±0.2
	2007	12	4368.1±1377.1	3952.1	2673.2-7267.8	14.4±0.4
	2008	11	3081.3±2059.4	2714.7	724.3-6458.5	14.4±0.2
	2009	10	3646.8±1395.5	3126.7	2162.8-6534.6	14.4±0.4
Ons	2004	15	771.4±496.7	754.1	121.9-1740.7	13.4±0.3
	2005	10	1785.3±818.2	1604.8	592.9-3804.7	13.5±0.3
	2006	12	1780.9±995.0	1846.7	268.1-3466.6	14.4±0.5
	2007	10	3402.1±1076.1	3059.3	2212.1-5353.4	14.6±0.5
Sagres	2004	15	1412.9±826.6	1168.7	357.7-3341.3	13.6±0.2
	2005	13	2355.5±925.6	2409.2	1268.1-4682.8	13.8±0.3
	2006	15	1797.6±464.5	1989.1	964.5-2520.1	14.4±0.3
	2007	10	4734.8±1036.5	5052.8	2524.7-6099.2	14.5±0.3

A more complex relationship among factors and covariables was apparent for Hg. The selected model accounted for colony, year and their interaction, and also for an effect of trophic level (Table 2). The interaction between colony and year (LR=16.9, d.f.=6, P =0.01) indicated there was no consistent spatial or temporal pattern. The significant effect of trophic level (LR=7.2, d.f.=1, P =0.008) indicated a common positive relationship with mercury concentration. Thus, the overall model showed that although the general trend with time was roughly similar among colonies (Figure 3), once adjusted for trophic level, the effect of year depended on the colony. For example, significant differences between 2004 and 2005 were clear only for Ons but not Cíes or Sálvora (Figure 3).

**Table 2:** Associated measures of information to a number of parameters of the different candidate models evaluated to fit the data corresponding to the trophic level and mercury concentration (log transformed).

**Model (Trophic level)**	**number of parameters**	**AICc**	***Δ*AICc**	**AICcWt**	**log-likelihood**
year	7	-273.32	20.07	<0.001	145.06
colony	3	42.51	335.89	<0.001	-17.15
**year+colony**	**9**	**-293.38**	**0.00**	**0.93**	**157.31**
year*colony	15	-288.21	5.17	0.07	161.69
*Current summer*					
CHL+SST+NAO	4	-43.16	250.22	<0.001	26.75
CHL+SST+NAO+colony	6	-39.19	254.19	<0.001	26.90
CHL*col + SST*col + NAO*col	12	-66.31	227.07	<0.001	47.20
*Previous spring*					
CHL+SST+NAO	4	-19.75	273.63	<0.001	15.05
CHL+SST+NAO+colony	6	-22.66	270.73	<0.001	18.66
CHL*col + SST*col + NAO*col	12	-83.02	210.36	<0.001	55.61
*Previous winter*					
CHL+SST+NAO	4	-150.65	142.73	<0.001	80.50
CHL+SST+NAO+colony	6	-158.40	134.98	<0.001	86.53
CHL*col + SST*col + NAO*col	12	-207.23	86.15	<0.001	117.72
*Previous autumn*					
CHL+SST+NAO	4	-152.69	140.69	<0.001	81.52
CHL+SST+NAO+colony	6	-150.68	142.70	<0.001	82.67
CHL*col + SST*col + NAO*col	12	-155.99	137.39	<0.001	92.10
**Model (Log Hg)**	**number of parameters**	**AICc**	***Δ*AICc**	**AICcWt**	**log-likelihood**
constant	1	159.22	173.32	<0.001	-77.58
colony	3	156.97	171.07	<0.001	-74.38
year	7	16.18	30.28	<0.001	0.31
year+colony	9	-5.05	9.05	0.01	13.14
year*colony	15	-9.40	4.70	0.06	22.28
TL	2	30.62	44.72	<0.001	-12.24
colony+TL	4	24.18	38.28	<0.001	-6.92
year+TL	8	2.58	16.68	<0.001	8.21
year+colony+TL	10	-11.30	2.80	0.15	17.40
year*colony+TL	**16**	**-14.10**	**0.00**	**0.60**	**25.84**
*Current summer*					
CHL+SST+NAO	4	82.34	96.45	<0.001	-36.01
CHL+SST+NAO+colony	6	76.71	90.81	<0.001	-31.04
CHL*col + SST*col + NAO*col	12	77.40	91.50	<0.001	-24.66
CHL+SST+NAO+TL	5	15.85	29.95	<0.001	-1.69
CHL+SST+NAO+colony+TL	7	7.82	21.93	<0.001	4.49
CHL*col + SST*col + NAO*col+TL	13	-5.06	9.05	0.01	17.74
*Previous spring*					
CHL+SST+NAO	4	97.45	111.56	<0.001	-43.55
CHL+SST+NAO+colony	6	83.59	97.70	<0.001	-34.47
CHL*col + SST*col + NAO*col	12	67.76	81.86	<0.001	-19.78
CHL+SST+NAO+TL	5	0.28	14.38	<0.001	6.10
CHL+SST+NAO+colony+TL	7	-10.54	3.56	0.10	13.70
CHL*col + SST*col + NAO*col+TL	13	-4.90	9.20	0.01	17.73
*Previous winter*					
CHL+SST+NAO	4	89.44	103.55	<0.001	-39.55
CHL+SST+NAO+colony	6	73.81	87.92	<0.001	-29.58
CHL*col + SST*col + NAO*col	12	30.46	44.56	<0.001	-1.13
CHL+SST+NAO+TL	5	17.85	31.95	<0.001	-2.68
CHL+SST+NAO+colony+TL	7	9.61	23.71	<0.001	3.62
CHL*col + SST*col + NAO*col+TL	13	-0.71	13.39	<0.001	15.64
*Previous autumn*					
CHL+SST+NAO	4	69.79	83.90	<0.001	-29.72
CHL+SST+NAO+colony	6	57.50	71.61	<0.001	-21.43
CHL*col + SST*col + NAO*col	12	23.11	37.21	<0.001	2.55
CHL+SST+NAO+TL	5	24.92	39.03	<0.001	-6.22
CHL+SST+NAO+colony+TL	7	14.14	28.25	<0.001	1.35
CHL*col + SST*col + NAO*col+TL	13	-10.01	4.10	0.08	20.28

Columns show the corrected Akaike Informacion criteria (AICc), the AICc increments (*Δ*AICc), Akaike weights (AICcWt) and log-likelihood of each candidate model.

**Figure 4:**
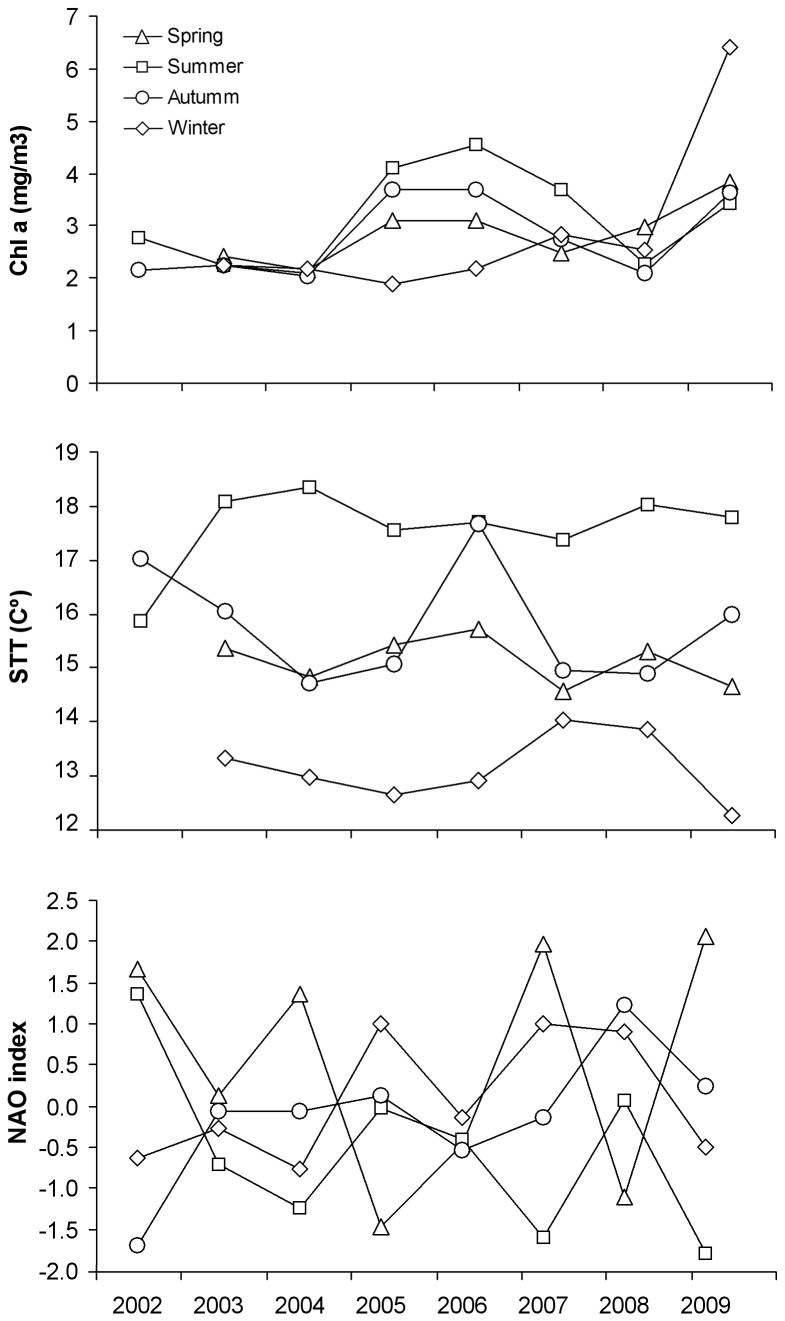
Chl a, SST and NOA index mean values for spring, summer, autumn and winter from 2002 to 2009.

We found no temporal significant differences in δ ^15^N of mussels sampled in Cíes (7.4 ± 0.6) indicating that there was no temporal change in the isotopic baseline of the wider region. Moreover, final models selected indicated that the environmental indices (Chl a, SST and NAO, see Table 2 and Figure 4) did not have any detectable direct or delayed effect on trophic level or mercury concentrations in shags, and instead that year was much more important.

## Discussion

Ideally, multiyear studies that include comparisons between impacted and reference areas have been recommended to assess recovery from environmental accidents or disturbances [47]. In our case, the spatial heterogeneity of oceanographic conditions and food web complexity of the NW coast of Spain [37–39,48], precluded the comparison between impacted and reference colonies. However, the three oil-impacted colonies monitored in this study reflected a similar temporal trend. Thus, our results showed that assessing trends simultaneously at several impacted sites can also be helpful for assessing long-term effects. Even though there were variations among their trophic levels and Hg concentrations, overall, shag chicks from the three colonies reflected an abrupt change in 2004 values and a gradual return to pre-spill levels reached in 2007 (Table 1; Figure 3). Such underlying temporal trend together with knowledge of potential prey from the same area (Figure 2 [27]) have allowed us not only to assess changes in trophic position of shags but also to relate them to specific changes in their feeding ecology. Both trophic level and Hg concentrations reflected that, after the wreck, shag chicks switched from a diet based on a high percentage of demersal and benthic fish (capelin and pout *Trisopterus* spp., corkwing wrasse *Symphodus melops*, ballan wrasse *Labrus bergylta* and gobies *Gobius* spp.) to a diet based on a higher proportion pelagic/semi-pelagic species (sand smelt *Atherina presbyter* and sandeels) and that it was not until 2007 that they return to pre-spill feeding ecology (Figure 3).

The oil spill could have affected the structure of the coastal food web and, consequently have given rise to the temporal changes in prey availability reflected on shag diet. Because of its heavy nature, the spilled oil in the *Prestige* accident was mainly stranded on the coast or sedimented in the form of oil patches [49]. As a consequence, several species of crustaceans, echinoderms and molluscs with a high sensitivity to oil exposure were affected by the petroleum [50]. Although little is known about its impact on fish population, shifts in the abundances of lower trophic level organisms as those just mentioned may have unleashed cascading bottom-up type ecosystem effects [5] reducing the abundance of benthic fish species and probably leading to a higher consumption of sand melt, one of the most abundant species of coastal pelagic fish in the waters off the Galician coast [51]. Moreover, top-down effects may also have been relevant due to spatio-temporal prohibitions on trawling following the *Prestige* oil spill [52] and affected the relative abundance of fish species. In the case of the *Prestige* accident, the closed areas affected not only the intertidal strip but also a large part of the continental shelf and therefore the fisheries for the area, both artisanal and industrial [52]. Such prohibitions may also have reduced fish mortality and affected the reproduction and survival of fish species and its abundance during the next year.

Although the bottom-up and/or top-down cascading effects described above may have affected simultaneously fish availability to top predators, the observed changes may be also explained by natural variability of environmental conditions. Climatic and oceanographic factors during the early life stages of fishes have long been implicated as a cause of recruitment fluctuations [33,53,54]. In this regard, a higher abundance of winter spawners such as sandeels has been positively related with the abundance of their plankton prey [34,36] and negatively related to winter NAO index and warm winters [34,35]. Consequently, lower winter NAO indices, lower winter temperatures and/or higher levels of primary production reflected in higher Chl a concentration in February-March may also explain a higher consumption of sandeels at summer 2004. Also fluctuations in primary production during the most important biological periods in the NW of Spain, including the spring and the summer blooms related to the upwelling of Eastern North Atlantic Central Waters may have affected the abundance of spring spawners such as wrasses [55]. However, there was no evidence that Chl a and SST changes were coincident with changes in shag feeding ecology (Table 2; Figure 4). Neither NAO index reflected any particular severity in environmental conditions for any year or season that may explain the abrupt change observed in shag diet (Table 2; Figure 4). Thus, temporal variation found in trophic level of chick feathers is more likely to be explained by long-lasting effects of the spilled oil than by the effect of any abrupt changes in climatic and oceanographic conditions.

It must be pointed out that our results concerning diet composition differ from those of Velando et al. [31], which showed a higher dependence on sandeels at Cíes during pre-spill years; however, differences in methodology preclude a direct comparison. Nevertheless, although most short-term studies published to date indicate a strong initial impact during the first year after the spill, with recovery by 2004 [50], our data confirm those of a previous contamination study [29], and highlight an impact on the Galician coastal marine ecosystem that lasted at least three years.

Previous work has shown that the high spatial and temporal variability in conditions on the NW Atlantic coast of Spain makes it difficult to differentiate anthropogenic impacts from natural environmental variation. Although several studies following the *Prestige* oil spill have tried to evaluate its effects on intermediate or low trophic-level species, the high background variability has made this a complex task [48,56,57]. Moreover, the direct monitoring of abundance of fish species characteristic of sandy and rocky bottoms in the main area affected by the *Prestige* oil would have been both expensive and technically challenging [58]. Although our study was limited to a single pre-spill data point, it was nevertheless possible to detect a longer-term impact. In this sense, monitoring of the feeding ecology of shags has proven to be an affordable and efficient way of detecting wider changes in the nearshore component of a Large Marine Ecosystem characterized by high oceanographic variability and food web complexity.

## Conclusions

Extensive long-term studies remain the only way to obtain consistent data allowing evaluation of anthropogenic impacts [35,59,60] and maintaining such studies should be a top priority for taking effective decisions about conservation, management and restoration actions. Aiming for a better global evaluation of pollution threats in the marine environment, the new European Marine Strategy Directive identified that the development of monitoring networks and suitable methodologies which could be applied to different marine ecoregions, was one of the most important current challenges for scientists [61]. This global approach comprises several tools such as the use of ecological indicators to assess the impact of human activities and to measure the response of marine ecosystems to anthropogenic disturbances. Our results showed how long-term impacts of oil spills can be assessed analysing alterations in relationships between prey and top predators using biochemical markers. This is therefore an efficient and affordable way of monitoring marine ecosystem health when collection of large-scale and long-term data covering all trophic levels is unfeasible due to limited funding or ecosystem complexity.
