# Correction: Ten Years after the *Prestige* Oil Spill: Seabird Trophic Ecology as Indicator of Long-Term Effects on the Coastal Marine Ecosystem

**DOI:** 10.1371/annotation/bb686276-234c-4881-bcd5-5051d0e66bfc

**Published:** 2013-10-23

**Authors:** Rocío Moreno, Lluís Jover, Carmen Diez, Francesc Sardà, Carola Sanpera

The name of the fourth author was incompletely given. The correct name is: Francesc Sardà-Palomera. The correct citation is: Moreno R, Jover L, Diez C, Sardà-Palomera F, Sanpera C (2013) Ten Years after the Prestige Oil Spill: Seabird Trophic Ecology as Indicator of Long-Term Effects on the Coastal Marine Ecosystem. PLoS ONE 8(10): e77360. doi:10.1371/journal.pone.0077360. The correct abbreviation in the Author Contributions statement is: FSP. 

